# The Examination of Prognostic Factors and Treatment Strategies for Traumatic Cerebrospinal Fluid Leakage

**DOI:** 10.7759/cureus.52874

**Published:** 2024-01-24

**Authors:** Kaoru Shizawa, Makoto Ohtake, Taisuke Akimoto, Takafumi Kawasaki, Shunsuke Seki, Yuya Imanishi, Masaki Yasuda, Takashi Kawasaki, Katsumi Sakata, Ichiro Takeuchi, Tetsuya Yamamoto

**Affiliations:** 1 Department of Neurosurgery, Yokohama City University Graduate School of Medicine, Yokohama, JPN; 2 Advanced Critical Care and Emergency Center, Yokohama City University Medical Center, Yokohama, JPN; 3 Department of Neurosurgery, Yokohama City University Medical Center, Yokohama, JPN

**Keywords:** multiple injuries, modified rankin scale, prognostic factors, surgical intervention, traumatic cerebrospinal fluid leakage

## Abstract

Introduction

This study aimed to determine the optimal timing for surgical intervention and the prognostic factors of cerebrospinal fluid (CSF) leakage.

Methods

We identified 25 patients with probable CSF leaks from 472 consecutive patients with head trauma. In addition to baseline characteristics and findings on admission, injury severity score (ISS), abbreviated injury score (AIS), and other factors related to CSF leakage were considered. We analyzed the prognostic factors after setting the primary endpoint as the modified Rankin Scale (mRS) at the time of discharge to determine the appropriate timing for surgical intervention.

Results

Univariate analysis revealed significantly poorer prognoses for elderly patients (p<0.001) and cases with low Glasgow Coma Scale (GCS) levels (p=0.039) and high D-dimer levels (p=0.028), which was consistent with findings from the analyses of all patients with head trauma. We found that multiple traumas (AIS≥3 at two or more sites, p=0.047) and high lactate levels (p=0.043) were poor prognostic factors specific to CSF leakage cases, while a longer time to CSF leakage cessation was also associated with a poorer prognosis (median, six days versus 13 days, p=0.014). An evaluation of the time to closure found that spontaneous cessation occurred within 14 days in most cases.

Conclusions

Conservative medical treatment is the first choice for most cases of traumatic CSF leakage. Surgical intervention should be considered if leakage does not cease after 14 days post injury. Furthermore, severe multiple injuries and high lactate levels were poor prognostic factors specific to patients with CSF leakage.

## Introduction

The existence of a cerebrospinal fluid (CSF) leak implies intracranial interchange with the paranasal sinuses or mastoid air cells, which can lead to meningitis and other intracranial infectious diseases. CSF leaks are typically classified as traumatic or non-traumatic, with traumatic cases being further classified as head injury- or surgery-related, depending on the etiology [1,2]. In patients with non-traumatic leaks, surgical closure is recommended as soon as a diagnosis of CSF leakage is made. Similarly, closure is recommended for leaks resulting from surgery-related trauma as soon as a diagnosis of CSF leakage is made. Endoscopic endonasal closure has been reported to improve the success rate of surgical closure in recent years [1,3,4].

The most common cause of traumatic CSF leakage is head trauma, especially from traffic injuries, with 1%-3% of head injuries resulting in CSF leakage [5]. CSF leakage is mainly associated with anterior basal skull fractures, usually occurring within 48 hours of injury, with 95% of leakages occurring within three months [1]. Friedman et al. [6] have reported that the rates of the spontaneous cessation of CSF leaks are high, occurring in 80%-95% of cases. Considering that the rate of spontaneous cessation is high, a conservative treatment approach is often adopted for CSF leakage in the acute phase following head injury. When conservative treatment fails to achieve closure or when spontaneous cessation is considered unlikely due to recurrence or late onset, a repair procedure is indicated. However, no global consensus exists on the appropriate timing of surgical intervention. Since spontaneous cessation occurs in most cases, studies on refractory cases are limited to case reports and case series with few reports on the specific timing of surgical indication and prognosis.

In this study, we conducted a retrospective examination of appropriate surgery timing and prognostic factors using imaging findings and clinical symptoms to identify probable cases of CSF leakage. We report, to the best of our knowledge, the first investigation into the specific timing of surgical intervention for patients with refractory CSF leakage.

## Materials and methods

Study design and participants

This study protocol was reviewed and approved by the Institutional Review Board and the Ethics Committee of the Yokohama City University Medical Center (approval number: B210400045). The requirement for written informed consent was waived, and individual informed consent was obtained via an opt-out approach, due to the retrospective nature of the study design and as per the Japanese Personal Information Protection Law and National Research Ethics Guidelines. The study procedures were performed in accordance with the ethical standards of the 1964 Declaration of Helsinki and its later amendments. This single-center, retrospective, cohort study included 25 patients diagnosed as probable CSF leakages from 472 consecutive patients with head trauma who were transported to our institute between January 2017 and October 2022. A diagnosis of probable CSF leakage was made only when fractures were visible on computed tomography (CT) imaging and the clinical evidence of serous leakage was apparent. Specifically, CT findings indicated fractures in areas of the skull that could cause CSF leakage, as well as poor air retention in the paranasal sinuses or mastoid air cells. In the clinical examinations, a diagnosis of CSF leakage was made when there was persistent serous fluid leakage from the nasal cavity or ear canal or fluid leakage into the retropharynx, which was determined by bronchoscopy. When it was unclear whether the leaking fluid was CSF, β-2 transferrin levels in the fluid were measured to confirm the diagnosis [7]. Suspected CSF leakage was diagnosed only if minor serous or pale blood fluid was observed from the nasal cavity or ear canal and if CSF leakage could not be ruled out completely. CSF leakage cessation was defined as the gross cessation of fluid leakage for 24 hours or more, including the cessation of serous fluid leakage from the nasal cavity or ear canal and the disappearance of retropharyngeal fluid leakage indicated by bronchoscopy.

Patient characteristics that were considered were age, gender, cause of injury, vital signs, pupil findings, blood test findings, injury severity score (ISS), revised trauma score (RTS), abbreviated injury score (AIS), a history of neurosurgical procedures (including CSF leak closure), the duration of CSF leakage, and time to the cessation of CSF leakage, and white blood cell (WBC) and C-reactive protein (CRP) levels during the CSF leakage period as signs of infection. The ISS is a severity rating method developed by Baker et al. [8] in 1974 for patients with multiple traumas, which is assessed using the anatomical aspects of multiple injury sites. After assigning injuries to six sites (the head and neck, face, chest, abdomen and pelvic organs, extremities and pelvis, and body surface), we noted the maximum AIS score for each site and then calculated the ISS as the sum of the squares of the maximum scores of the top three sites. Furthermore, RTS, as calculated from the Glasgow Coma Scale (GCS), systolic blood pressure, and respiratory rate, was used as a physiological index of severity and an endpoint. In this study, the ISS was used as an indicator of severe trauma, with an ISS score of ≥16 defined as severe trauma, and the AIS as an indicator of multiple traumas, with an AIS score of ≥3 in two or more sites defined as multiple traumas. The probability of survival, which was derived from AIS and RTS, was excluded from the analysis due to confounding relationships.

Treatment protocol

A diagnosis of CSF leakage was made based on clinical findings and cranial CT at presentation. Patients with probable CSF leakage were assigned bed rest in the semi-Fowler’s position (15-30 degrees) and continuously monitored for CSF leakage every few hours over consecutive days. For patients with severe CSF leakage, prophylactic antibiotic therapy, primarily ceftriaxone, was administered. The cases that required early surgical intervention, such as those involving facial bone fractures or requiring craniotomy to remove hematomas, were excluded from the assessment of spontaneous cessation due to the simultaneous surgical closure of the CSF leak. According to our institution’s policy, the risk of meningitis infection associated with negative pressure is acknowledged, and CSF testing and drainage are not performed in the absence of any signs of meningitis. Except for one case, the cases were managed without continuous CSF drainage. CSF drainage was included in conservative treatment. For CSF leak closure intervention, endoscopic endonasal closure or direct closure with craniotomy was chosen depending on the site of the leak once located using CT or magnetic resonance imaging [4,9]. During surgical intervention, although the direct confirmation of dural damage was required, if direct confirmation was not possible due to deep localization, closure techniques such as covering with DuraSeal (Integra LifeSciences, Plainsboro, NJ), a synthetic, absorbable polyethylene glycol (PEG) hydrogel, were performed for dura closure.

Outcome measurement

The primary endpoint was the modified Rankin Scale (mRS) score at the time of discharge. The time to the spontaneous cessation of CSF leakage or surgical intervention was evaluated using the Kaplan-Meier curve. For the prognostic evaluation, the good prognosis group consisted of cases showing an mRS score of 0-2, while the poor prognosis group consisted of cases with an mRS score of 3-6.

Statistical analysis

To reduce bias, the analysis was performed after the completion of the study. The results are presented as the mean and standard deviation for quantitative data and frequencies (percentages) for categorical data. Data were not normalized due to the limited number of enrolled patients. For comparisons between groups, Pearson’s chi-square test (or Fisher’s exact test) and the Wilcoxon test were performed. Kaplan-Meier analyses were performed to evaluate the time to CSF leak cessation. Statistical significance was set at p<0.05. All statistical analyses were performed using JMP 15 (SAS Institute Inc., Cary, NC).

## Results

**Figure 1:**
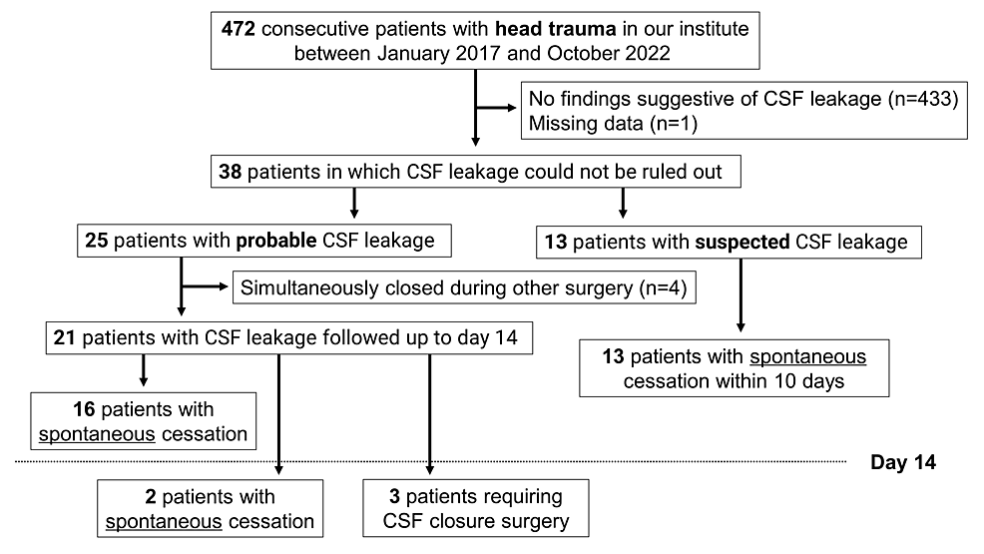
Summary of the 472 cases CSF: cerebrospinal fluid

A summary of the 472 cases is shown in Figure 1. An overview of patient characteristics from the 25 probable CSF leakage cases is shown in Table 1. The average age was 43.8±27.2 (2-88) years, 22 (88.0%) patients were male, 12 (48.0%) sustained traffic injuries, eight (32.0%) sustained fall-related injuries, and five (20.0%) sustained crash-related injuries. Seventeen cases (68.0%) involved severe trauma (ISS≥16), 13 (52.0%) involved multiple injuries (AIS≥3 in more than two sites), and 17 (68.0%) presented with a GCS of ≥13 at the time of hospitalization. A total of 11 (44.0%) cases involved a history of neurosurgical procedures; of these, the surgical closure of CSF leakage where spontaneous cessation did not occur was performed in three cases (12.0%). Meningitis was diagnosed in only one patient that required lumbar drainage, based on blood and CSF pathology findings. The overall time to the cessation of CSF leakage was 10.7±9.1 (1-38) days, and 14 patients (56.0%) were in the good prognosis group with mRS scores of 0-2 at the time of discharge. No patients died while in the hospital.

**Table 1:** Patients characteristics GCS, Glasgow Coma Scale; ISS, injury severity score; AIS, abbreviated injury score; CSF, cerebrospinal fluid; mRS, modified Rankin Scale; eTSS, endoscopic trans-sphenoidal surgery; M, male; F, female; N/A, not available

Number	Age	Sex	Trauma mechanisms	Type of leakage	GCS	ISS	AIS (head)	Other sites of AIS 3 or higher	D-dimer (µg/mL)	Lactate (mmol/L)	Time to CSF cessation (days)	Type of CSF cessation	Reason for surgical intervention	mRS at discharge
1	22	M	Traffic	Rhinorrhea	11	24	4	None	31.4	1.1	8	Craniotomy	Simultaneous with intermaxillary fixation	1
2	49	F	Crash	Rhinorrhea	3	9	3	None	25.3	4.8	13	Spontaneous	-	4
3	52	M	Fall	Rhinorrhea	15	13	2	Face	4.2	1.5	7	Craniotomy	Simultaneous with facial bone reconstruction	0
4	47	M	Traffic	Rhinorrhea	13	18	3	Pelvis	34	0.6	13	Spontaneous	-	3
5	2	M	Traffic	Otorrhea	14	5	2	None	N/A	N/A	3	Spontaneous	-	0
6	76	F	Traffic	Otorrhea	14	27	3	Chest and pelvis	37.8	3.1	7	Spontaneous	-	3
7	28	M	Traffic	Rhinorrhea	8	29	4	Face	61.2	4.7	30	Craniotomy	CSF closure (anterior skull base)	5
8	20	M	Traffic	Rhinorrhea	14	24	4	None	47.5	2	4	Spontaneous	-	2
9	88	F	Crash	Rhinorrhea	3	34	4	Chest and pelvis	55.5	8.7	3	Spontaneous	-	5
10	20	M	Traffic	Rhinorrhea	6	26	4	Face	35.4	19	3	Spontaneous	-	1
11	26	M	Fall	Rhinorrhea	11	29	4	Chest	29.2	1.9	18	Spontaneous	-	2
12	67	M	Traffic	Otorrhea	8	17	4	None	207.8	4.2	23	Spontaneous	-	5
13	8	M	Fall	Rhinorrhea	9	9	3	None	33.4	4	4	Craniotomy	Simultaneous with facial bone reconstruction	1
14	61	M	Crash	Rhinorrhea	15	13	3	None	15.7	2.1	1	Craniotomy	Simultaneous with subdural hematoma removal	0
15	88	M	Fall	Rhinorrhea	13	22	3	Pelvis	64.7	5	4	Spontaneous	-	4
16	53	M	Fall	Rhinorrhea	14	17	3	None	4.8	1.2	8	Spontaneous	-	1
17	22	M	Traffic	Rhinorrhea	3	35	5	Face	34.4	3.9	13	Spontaneous	-	3
18	81	M	Crash	Rhinorrhea	6	9	2	Abdomen	226	2.9	18	eTSS	CSF closure (clivus)	5
19	61	M	Crash	Otorrhea	12	22	3	Chest	21.6	4.4	5	Spontaneous	-	3
20	59	M	Traffic	Otorrhea	14	29	4	Face	11.8	4.1	14	Spontaneous	-	4
21	16	M	Traffic	Rhinorrhea	15	21	4	None	15.7	1	3	Spontaneous	-	1
22	13	M	Fall	Otorrhea	13	9	3	None	33.8	2.5	3	Spontaneous	-	1
23	85	M	Fall	Otorrhea	6	9	3	None	51.6	21	38	Craniotomy	CSF closure (temporal bone with ear canal injury)	5
24	30	M	Fall	Rhinorrhea	12	34	4	Chest and pelvis	63.7	16	12	Spontaneous	-	1
25	19	M	Traffic	Rhinorrhea	7	24	4	None	11.6	3.4	12	Spontaneous	-	2

**Figure 2:**
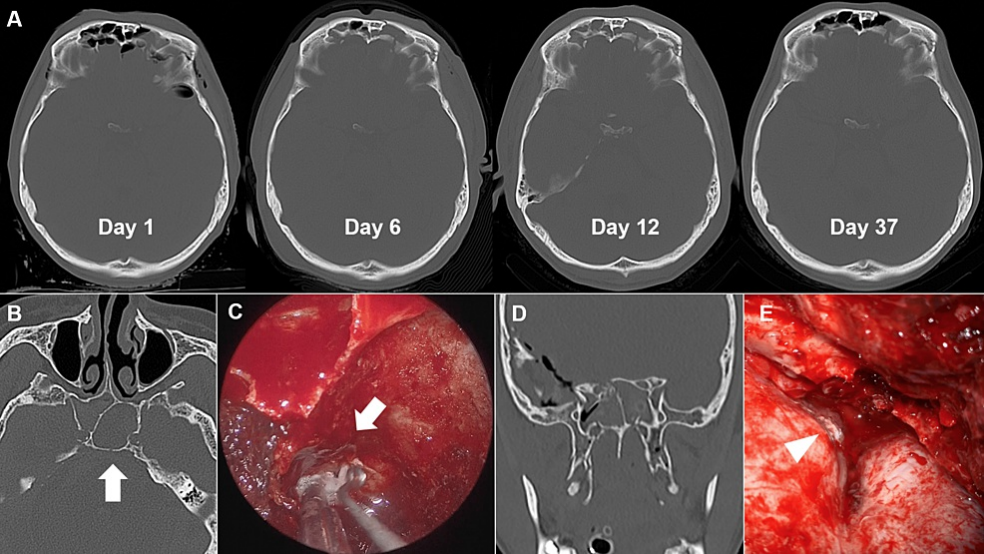
Imaging findings (A) Computed tomography (CT) scan of the brain revealing changes in air content in the frontal sinus over time, (B) CT scan showing a fracture of the clivus (arrow), (C) an intraoperative view of endoscopy showing fat filling in the cerebrospinal fluid (CSF) leak site of the clivus (arrow), (D) CT coronal view showing compound fractures of the anterior skull base, and (E) intraoperative view showing a site of dural injury in the anterior skull base leaking CSF (arrowhead).

Figure 2A (case 24) depicts the development of cranial CT findings leading up to spontaneous cessation with conservative treatment. The patient sustained multiple skull fractures extending from the anterior basal skull to the frontal sinus, with persistent CSF leakage from the nose. As a result of conservative follow-up, the visible CSF leakage disappeared on day 12, and CT showed that the fluid accumulation in the frontal sinus gradually washed out over subsequent days. Figure 2B, 2C (case 18) shows a patient with persistent CSF rhinorrhea, which required a trans-nasal endoscopic CSF leak closure on day 18. Cranial CT confirmed a fracture of the clivus, and a CSF leak corresponding to the fracture was found intraoperatively and closed by infilling with abdominal fat. Figure 2D, 2E (case 7) shows a case of surgical closure via craniotomy. As shown in the images, in cases where spontaneous cessation did not occur, multiple dural tears were identified intraoperatively.

Table 2 shows the univariate and multivariate analyses of the prognostic factors of 471 head trauma cases, excluding one case with missing data. In the multivariate analysis of the prognosis, age (p<0.001), low GCS (p<0.001), and D-dimer levels (p=0.019) were independent associated factors. Out of the 25 CSF leakage cases, Table 3 shows the results of the analysis of prognostic factors for the good prognosis group. While the number of patients is small and only univariate analysis was performed, prognoses were significantly worse for patients of advanced age (p<0.001) and those with low GCS (p=0.039) and high D-dimer levels (p=0.028). Furthermore, patients with multiple traumas with concomitant CSF leakage (more than two sites with an AIS of ≥3, p=0.047) and high lactate levels (p=0.043) had significantly poorer prognoses. However, while a surgical history of CSF leak closure or a high inflammation change during CSF leakage did not affect prognosis, a longer time to CSF leakage cessation was associated with a poorer prognosis (median, six (1-18) days versus 13 (3-38) days, p=0.014).

**Table 2:** Univariate and multivariate analysis of prognostic factors in 471 patients with head trauma Data are presented as the number (%), the mean±standard deviation (SD), or the median (range) *Unit odds ratio GCS, Glasgow Coma Scale; ISS, injury severity score; RTS, revised trauma score; AIS, abbreviated injury score; mRS, modified Rankin Scale; WBC, white blood cell; RBC, red blood cell

				Univariate	Multivariate	
	Total (n=471)	mRS 0-2 (n=277)	mRS 3-6 (n=194)	P value	Odds ratio (95% CI)	P value
Mean age±SD (years)	49.4±24.7	39.8±26.3	63.0±22.3	<0.001	1.04 (1.03-1.05)*	<0.001
Sex male (%)	345 (73.3)	204 (73.7)	141 (72.7)	0.816	-	-
Skull fracture (%)	215 (46.1)	123 (45.1)	92 (47.7)	0.577	-	-
Neurosurgical surgery (%)	151 (32.1)	66 (23.8)	85 (43.8)	<0.001	1.46 (0.86-2.48)	0.156
Non-neurosurgical surgery (%)	60 (12.7)	34 (12.3)	26 (13.4)	0.718	-	-
GCS mean±SD	10.1±4.2	11.6±3.9	8.0±4.5	<0.001	0.84 (0.78-0.90)*	<0.001
Anisocoria (%)	37 (7.9)	10 (3.6)	27 (13.9)	<0.001	2.17 (0.80-5.88)	0.126
Bilateral mydriasis (%)	67 (14.2)	26 (9.4)	41 (21.1)	<0.001	1.28 (0.59-2.78)	0.534
ISS mean±SD	19.4±10.6	17.6±10.3	21.9±11.0	<0.001	1.01 (0.98-1.03)*	0.652
RTS mean±SD	6.5±1.8	7.1±1.4	5.7±2.3	<0.001	1.02 (0.86-1.20)*	0.850
AIS≧3 at two or more sites (%)	109 (23.1)	60 (21.7)	49 (25.3)	0.362	-	-
Blood test results at the time of visit						
WBC mean±SD (×10^3^/µL)	12.5±5.6	12.7±5.4	12.3±5.8	0.283	-	-
RBC mean±SD (×10^4^/µL)	4.1±0.7	4.2±0.7	4.0±0.8	<0.001	1.66 (0.91-3.65)*	0.163
Platelet mean±SD (×10^3^/µL)	230.8±88.3	248.4±90.5	205.5±85.0	<0.001	1.00 (0.99-1.00)*	0.898
Hemoglobin mean±SD (g/dL)	12.8±3.9	13.2±4.6	12.2±2.5	0.0031	0.84 (0.66-1.00)*	0.126
D-dimer median (range) (µg/mL)	24.6 (0.5-951.2)	14.7 (0.5-581.5)	42 (1-951.2)	<0.001	1.00 (1.00-1.98)*	0.019
Fibrinogen median (range) (µg/mL)	243 (51-832)	243.5 (51-630)	239 (55-832)	0.348	-	-
Lactate median (range) (mmol/L)	2.5 (0.5-100)	2.3 (0.6-100)	2.9 (0.5-79.7)	<0.001	0.99 (0.96-1.02)*	0.745
Creatinine median (range) (mg/dL)	0.77 (0.1-9.53)	0.71 (0.1-9.53)	0.875 (0.16-7.45)	<0.001	1.35 (1.02-1.97)*	0.062

**Table 3:** Univariate analysis of prognostic factors in 25 patients with CSF leakage Data are presented as the number (%), the mean±standard deviation (SD), or the median (range) †Fisher’s exact test *There were one missing data and 12 in the good prognosis group GCS, Glasgow Coma Scale; ISS, injury severity score; RTS, revised trauma score; AIS, abbreviated injury score; CSF, cerebrospinal fluid; mRS, modified Rankin Scale; WBC, white blood cell; RBC, red blood cell; CRP, C-reactive protein

	Total (n=25)	mRS 0-2 (n=13)	mRS 3-6 (n=12)	P value
Mean age±SD (years)	43.8±27.2	26.3±18.1	62.6±22.6	<0.001
Male sex (%)	22 (88.0)	13 (100)	9 (75.0)	0.096†
Skull fracture (%)	25 (100)	16 (100)	9 (100)	-
Neurosurgical surgery (%)	12 (48.0)	7 (53.9)	5 (41.7)	0.695†
Non-neurosurgical surgery (%)	3 (12.0)	1 (7.7)	2 (16.7)	0.593
GCS mean±SD	10.4±4.0	12.0±3.1	8.5±4.4	0.039
Anisocoria (%)	3 (12.0)	2 (15.4)	1 (8.3)	0.999†
Bilateral mydriasis (%)	4 (16.0)	3 (23.1)	1 (8.3)	0.593†
ISS mean±SD	20.3±8.8	19.1±8.8	21.7±9.5	0.477
RTS mean±SD	6.4±2.0	7.3±0.9	5.5±2.2	0.025
AIS≧3 at two or more sites (%)	11 (44.0)	3 (23.1)	8 (66.7)	0.047†
Blood test results at the time of visit				
WBC mean±SD (×10^3^/µL)	14.5±6.1	14.8±4.5	14.3±8.0	0.341
RBC mean±SD (×10^4^/µL)	4.4±0.8	4.7±0.5	4.1±1.0	0.087
Platelet mean±SD (×10^3^/µL)	241±70	254±55	226±86	0.211
Hemoglobin mean±SD (g/dL)	13.5±1.8	14.0±1.4	12.8±2.1	0.108
D-dimer median (range) (µg/mL)	33.9 (4.2-226)	30.3 (4.2-63.7)*	44.7 (11.8-226)	0.028
Fibrinogen median (range) (µg/mL)	234 (105-341)	253 (147-341)	226.5 (105-278)	0.174
Lactate median (range) (mmol/L)	3.7 (0.6-21)	2.1 (1-19)*	4.3 (0.6-21)	0.043
Creatinine mean±SD (mg/dL)	0.86±0.34	0.80±0.32	0.93±0.38	0.399
Items related to CSF leakage				
CSF leak closure surgery (%)	7 (28.0)	4 (30.7)	3 (25.0)	0.711†
Duration of CSF leakage median (range) (days)	8 (1-38)	6 (1-18)	13 (3-38)	0.014
Blood test results during the CSF leak period				
Maximum WBC mean±SD (×103/µL)	14.7±5.5	13.3±4.3	16.1±7.8	0.446
Maximum CRP mean±SD (mg/dL)	9.4±5.0	8.2±5.6	10.8±4.7	0.142

**Figure 3:**
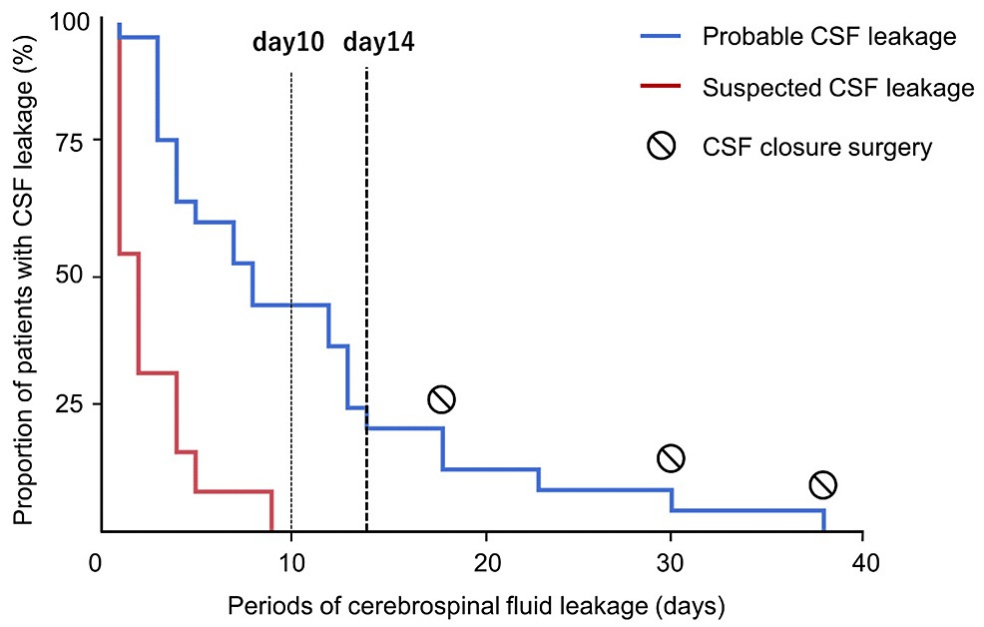
Kaplan-Meier curve of time to cerebrospinal fluid (CSF) leak cessation in the patients in the probable CSF leakage group and suspected CSF leakage group

The time to spontaneous cessation or surgical intervention is shown as a Kaplan-Meier curve. We identified 13 patients with suspected CSF leakage from patients that could not be diagnosed with probable CSF leakage as defined in this study but with fluid leakage from the nose or ears where CSF leakage could not be ruled out. In all suspected cases, visible spontaneous cessation occurred within 10 days (Figure 3). However, in the 18 cases (72.0%) of spontaneous cessation out of the 25 probable cases of CSF leakage, leakage was resolved within 14 days in 16 cases (88.9%), and spontaneous cessation occurred when closure surgery was considered after 14 days or more in two cases (11.1%). Of the five cases involving continued leakage for more than 14 days, three (60.0%) required surgical closure. No significant differences in background, clinical findings, and test results were found between the three cases requiring surgical closure and the cases with spontaneous cessation.

## Discussion

In cases of CSF leakage, the timing of surgical closure is difficult to determine because spontaneous cessation occurs in the majority of CSF leakage cases. CSF leakage treatment primarily aims to stop the leak and prevent meningitis. Conservative treatment is often the first choice as most traumatic cases heal spontaneously. Furthermore, considering that the occurrence of refractory traumatic CSF leaks is low, no large-scale global analyses have been conducted. Consequently, little evidence exists regarding the appropriate timing and prognosis of surgical intervention. To the best of our knowledge, this is the first report that analyzes the specific timing of surgical intervention for patients with refractory CSF leakage.

Traumatic CSF leakage is the intracranial leakage of spinal fluid resulting from trauma, which typically appears within several days following injury and usually resolves spontaneously. However, in a fraction of patients, refractory or recurrent meningitis may ensue for which treatment can be exceedingly difficult [10,11]. Bacterial meningitis is the most serious complication of CSF leakage, which may lead to neurological sequelae and can even be fatal. Despite several studies on the usefulness of continuous CSF drainage and the prophylactic administration of antimicrobial agents as conservative treatment strategies, a consensus on standard treatment policy has not yet been established [2,10,12]. Reducing intracranial pressure via continuous CSF drainage is effective in closing CSF leakage, but caution is warranted if an infection is present in the nasal sinuses as negative pressure increases the possibility of the intracranial seeding of the infection. The standard policy at our institution is to avoid continuous drainage of CSF because of the difficulty experienced in treating refractory meningitis that develops after several days of continuous CSF drainage. Patients must be fully informed that they are strictly prohibited from blowing their noses. Close follow-up, taking into account the fever type and inflammation findings (WBC and CRP levels and CSF examination), is necessary. In our study, CSF testing was performed in eight of the 25 patients, and meningitis was diagnosed in only one patient (case 2) based on a positive culture and a significant reduction in the CSF glucose level.

While meningitis occurs in approximately 7%-30% of patients with CSF leakage, no consensus exists on the suitability of prophylactic antibiotic agent administration. The incidence of meningitis is proportional to the severity and duration of CSF leakage [10-12]. Although some studies have reported a definite reduction in the frequency of meningitis with the administration of antibiotic agents, others have reported no change [11,13]. In patients considered to be at high risk of developing meningitis, such as patients with severe CSF leakage, we administer a broad-spectrum antibiotic agent with good CSF transferability (third-generation cephem). Of the 25 patients included here, only one patient (4%, case 2) required lumbar drainage and presented with meningitis requiring additional treatment. Therefore, we believe that antibiotic agents prevent meningitis to a certain degree. Brodie and Thompson [14] have reported the risk of meningitis complications following spontaneous cessation. The dura mater does not regenerate spontaneously and can only close by the regeneration of a single layer of fibrous connective tissue or nasal mucosa. Long-term follow-up after spontaneous closure indicates that 30%-40% of patients develop late-onset meningitis; therefore, families should be informed of the risk of late-onset meningitis even if the CSF leak has closed [1].

Regarding the timing of the spontaneous cessation of traumatic CSF leakage, some reports [1,5,15] have revealed that cessation occurs within 1-3 weeks in 50%-80% of patients with rhinorrhea and 5-10 days in 80%-85% of those with otorrhea. Of the 25 cases, the spontaneous cessation of CSF leakage could not be followed up until day 14 in four cases due to the early closure of CSF leakage simultaneous with other surgeries, such as facial bone reconstruction. In the remaining 21 cases of probable CSF leakage, spontaneous cessation occurred in 57.1% (8/14) of nasal leakage cases and 85.7% (6/7) of ear leakage cases after an average of 8.9±3.2 (3-18) days and 9.2±4.5 (3-23) days, respectively, showing slightly earlier spontaneous cessation than that in previous reports, especially in the rhinorrhea cases. We also found that in most cases in which spontaneous cessation occurred, it occurred within 14 days (16/18, 88.9%). In addition, in all cases involving suspected CSF leakage, the leakage spontaneously resolved within 10 days, suggesting the feasibility of conservative treatment for 14 days post injury with the goal of spontaneous cessation. In contrast, as our analysis showed a trend toward increasingly poorer prognoses as the time to spontaneous cessation increased (p=0.014), we cannot recommend long-term conservative treatment with the expectation of spontaneous cessation. In our study, of the five cases in which CSF leakage continued for more than 14 days, three (60.0%) required surgical closure. As no significant differences in all characteristics were observed between the three cases in which surgical closure was required and the cases of spontaneous cessation, the prediction of the need for surgical intervention was considered difficult. Therefore, we believe that opting for surgical intervention based on the number of days after injury is important. These results suggest that traumatic CSF leakage can be treated conservatively for up to 14 days with an expectation of spontaneous cessation. However, for cases in which leakage continues beyond the 14th day, we recommend considering surgical closure because of the negative impact of prolonged CSF leakage on prognosis. Nasal sinus scintigraphy and endonasal endoscopy with the intrathecal injection of a fluorescent dye have been reported in cases where the leakage site location is unknown and the careful evaluation of indications is required due to the risk of complications from intracranial infection [3,5].

Consistent with previous studies [16,17], in our analysis of all patients with head trauma, advanced age, low GCS level, and high D-dimer level were associated with significantly poorer prognoses. The analysis of patients with CSF leakage also indicated significantly poorer prognoses for patients with advanced age, low GCS levels, and high D-dimer levels, but we do not believe that this finding is unique to CSF leakage cases. Evaluating the difference in analysis results between all cases and CSF leakage cases, multiple trauma cases with severe truncal injuries in combination with head injuries and high lactate levels were specific poor prognosis factors for CSF leakage cases, with high lactate levels considered to be associated with circulatory failure due to trunk injury. These adverse effects are assumed to be due to the difficulty in maintaining rest following severe truncal injury and the stimulation caused by multiple trunk examinations and therapeutic interventions. For patients with CSF leakage coinciding with truncal injury, early-stage surgical intervention to stop CSF leakage may be effective in improving their overall condition.

Limitations

Very few reports on refractory traumatic CSF leaks exist, and a sufficient literature review has not yet been conducted. Since our institution primarily treats severe trauma, we encounter a relatively large number of patients with multiple traumas; however, only a few patients could be diagnosed as probable CSF leakages, and only univariate analysis could be performed. Our treatment policy does not include CSF drainage, which is considered one of the effective treatment methods, and is considered to be a treatment bias in this clinical trial. Considering that surgical intervention was performed in only a small number of cases, we believe that this suggests that spontaneous cessation can be expected in most cases. While refractory CSF leakage is infrequent, its occurrence can lead to very poor outcomes, leading us to believe that continuing the gathering of evidence on its treatment is crucial. In the future, we hope to conduct multi-site prospective studies and investigations that produce high-level evidence.

## Conclusions

In conclusion, the coincidence of severe truncal injury (AIS≥3 at more than two sites) is considered a poor prognostic factor specific to cases of CSF leakage. Furthermore, since spontaneous cessation can be expected in the majority of cases, conservative treatment is the first choice for most traumatic CSF leaks. However, since the prognosis worsens over the duration of the leak, surgical intervention should be recommended more than 14 days post injury.
